# Adverse In-Hospital Outcomes Following Robot-Assisted vs. Open Radical Prostatectomy in Quadragenarians

**DOI:** 10.3390/cancers17071193

**Published:** 2025-03-31

**Authors:** Fabian Falkenbach, Francesco Di Bello, Natali Rodriguez Peñaranda, Mattia Longoni, Andrea Marmiroli, Quynh Chi Le, Calogero Catanzaro, Michele Nicolazzini, Zhe Tian, Jordan A. Goyal, Nicola Longo, Stefano Puliatti, Riccardo Schiavina, Carlotta Palumbo, Gennaro Musi, Felix K. H. Chun, Alberto Briganti, Fred Saad, Shahrokh F. Shariat, Gisa Mehring, Lars Budäus, Markus Graefen, Pierre I. Karakiewicz

**Affiliations:** 1 Cancer Prognostics and Health Outcomes Unit, Division of Urology, University of Montreal Health Center, Montreal, QC H2X 0A9, Canada; 2 Martini-Klinik Prostate Cancer Center, University Medical Center Hamburg-Eppendorf, 20246 Hamburg, Germany; 3 Department of Neurosciences, Science of Reproduction and Odontostomatology, University of Naples Federico II, 80138 Naples, Italy; 4 Department of Urology, Ospedale Policlinico e Nuovo Ospedale Civile S. Agostino Estense Modena, University of Modena and Reggio Emilia, 41126 Modena, Italy; 5 Division of Experimental Oncology/Unit of Urology, IRCCS Ospedale San Raffaele-Vita-Salute San Raffaele University, 20132 Milan, Italy; 6 Department of Urology, IEO European Institute of Oncology, IRCCS, 20141 Milan, Italy; 7 Department of Oncology and Haemato-Oncology, Università degli Studi di Milano, 20122 Milan, Italy; 8 Department of Urology, University Hospital, Goethe University Frankfurt, 60590 Frankfurt am Main, Germany; 9 Division of Urology, IRCCS Azienda Ospedaliero-Universitaria di Bologna, 40138 Bologna, Italy; 10 Division of Urology, Department of Translational Medicine, Maggiore della Carità Hospital, University of Eastern Piedmont, 28100 Novara, Italy; 11 Division of Urology, Department of Oncology, University of Turin, 10124 Orbassano, Italy; 12 Department of Urology, Comprehensive Cancer Center, Medical University of Vienna, 1090 Vienna, Austria; 13 Department of Urology, Weill Cornell Medical College, New York, NY 10065, USA; 14 Department of Urology, University of Texas Southwestern Medical Center, Dallas, TX 75390, USA; 15 Hourani Center for Applied Scientific Research, Al-Ahliyya Amman University, Amman 19111, Jordan; 16 Department of Urology, University Medical Center Hamburg-Eppendorf, 20246 Hamburg, Germany

**Keywords:** prostatectomy, prostate cancer, young patients, NIS

## Abstract

**Background/Objectives**: Adverse in-hospital outcomes at radical prostatectomy have not been specifically addressed in young patients aged 40–49 years (quadragenarians). Additionally, no comparison between robot-assisted (RARP) vs. open radical prostatectomy (ORP) has been reported in this population. **Methods**: Descriptive analyses, propensity score matching (PSM), and multivariable logistic/Poisson regression models addressed quadragenarians undergoing RARP or ORP within the National Inpatient Sample (2009–2019). **Results**: Of 5426 quadragenarians, 4083 (75.2%) and 1343 (24.8%) underwent RARP and ORP, respectively. The proportion of RARP increased from 68.1 to 84.5% (2009–2019, EAPC: +2.8%, *p* < 0.001). Adverse in-hospital outcomes after RARP were invariably lower than those after ORP. Specifically, the rates of overall complications (7.8 vs. 13.4%, Δ −5.6%, multivariable odds ratio (OR): 0.54), blood transfusions (1.2 vs. 6.3%, Δ −5.1%, OR: 0.21), and length of stay (LOS) > 2 days (10.6 vs. 28.7%, Δ −18.1%, OR: 0.32) were lower after RARP than after ORP (all *p* < 0.001). After additional one-to-one PSM between ORP and RARP patients, virtually the same results were reported (overall complications: 7.0 vs. 13.4%, Δ −6.4%, OR: 0.49; blood transfusion rates: 1.5 vs. 6.3%, Δ −4.8%, OR: 0.23; LOS > 2 days: 10.9 vs. 28.7%, Δ −17.8%, OR: 0.30). Conversely, RARP use resulted in higher total hospital charges (USD 43,690 vs. 36,840, Δ USD +6850, IRR: 1.18; *p* < 0.001). **Conclusions**: Quadragenarians exhibited a more favorable adverse in-hospital outcome profile after RARP vs. ORP. These advantages are offset by a small, albeit significant, increase in total hospital charges.

## Simple Summary

Young patients with prostate cancer before the age of 50 years represent a special patient population at radical prostatectomy. Besides higher baseline urinary and erectile function, longer life expectancy, and different cancer phenotypes, patients in their 40s are also often at the peak of their careers and may have young families. Therefore, the potential impact of adverse in-hospital outcomes may be particularly devastating for these patients and their families. We analyzed the largest cohort of more than 5000 such patients using a national representative registry. We found that more young men were getting robot-assisted prostate cancer surgery instead of open surgery over time. Surgery using a robot led to fewer problems in the hospital and shorter hospital stays. The biggest advantages of the robot were seen concerning blood transfusions and overall complications, while the advantages of the robot were modest in most other comparison categories. Conversely, using the robot costed a little bit more than open surgery.

## 1. Introduction

Prostate cancer (PCa) is relatively rarely diagnosed in patients under the age of 50 years [1,2,3]. In consequence, data regarding treatment outcomes of clinically localized PCa in patients aged 40–49 are scarce [4,5,6,7,8,9,10,11,12,13]. Specifically, differences in adverse in-hospital outcome profiles between robot-assisted (RARP) and open radical prostatectomy (ORP) of these individuals are unknown. No formal head-to-head comparisons have been reported on this topic. However, small-scale studies have recorded exceedingly low complication rates in quadragenarians for both RARP and ORP separately [5,11]. Therefore, ORP may remain an equally effective treatment alternative for young patients concerning adverse in-hospital outcomes [14]. We hypothesized that RARP and ORP exhibit the same adverse in-hospital outcome profiles in quadragenarians. Additionally, we tested whether differences in total hospital charges (THC) distinguish RARP from ORP in this patient population. We tested this hypothesis in a contemporary, population-based, and large-scale cohort of quadragenarians treated with RP within the National Inpatient Sample (NIS) from 2009 to 2019.

## 2. Materials and Methods

### 2.1. Data Source

We relied on discharge data from the NIS (2009–2019) to test for adverse in-hospital outcomes after RP. The longitudinal NIS databases are included in the Healthcare Cost and Utilization Project (HCUP) by the Agency for Healthcare Research and Quality (AHRQ) within a Federal-State partnership [15]. The International Classification of Disease (ICD) 9th revision Clinical Modification (ICD-9-CM), ICD 10th revision Clinical Modification (ICD-10-CM), and the ICD 10th revision Procedure Coding System (ICD-10-PCS) were applied for coding diseases, outcomes, and procedures.

### 2.2. Study Population

We focused on patients aged 40 to 49 years (quadragenarians) with a primary diagnosis of PCa (ICD-9-CM codes 185, 233.4, 236.5, V10.46; ICD-10-CM codes C61, D07.5, D40.0, Z85.46). Only patients treated with RP (RARP or ORP) were included, in accordance with a previously established methodology [16]. Patients were stratified according to the surgical approach (RARP vs. ORP).

### 2.3. Outcomes of Interest

The endpoints consisted of adverse in-hospital outcomes, defined as complications (overall, intraoperative, bleeding, cardiac, respiratory, genitourinary, wound, infectious, vascular, miscellaneous surgical, miscellaneous medical), rates of blood transfusions, critical care therapy use, prolonged length of stay (LOS; >2 days), and in-hospital mortality, identified by ICD-9 and ICD-10 codes according to a previously reported methodology. Total hospital charges (THC) were directly obtained from the NIS and were based on hospital accounting reports in accordance with the NIS methodological guidelines [15]. Within the NIS, THC is adjusted to the 2016 United States dollar (USD), and their definition relies on the overall Consumer Price index by the US Bureau of Labor Statistics. The Deyo modification of the Charlson Comorbidity Index (CCI) was applied to account for comorbidities [17]. We relied on the coding algorithms for defining comorbidities using ICD-9-CM and ICD-10-CM codes by Quan et al. [18]. The covariables included age at admission (years, continuously coded), ethnicity (Caucasian vs. Afro-American vs. others), CCI (0 vs. 1 vs. ≥2), pelvic lymph node dissection (PLND, yes vs. no), year of surgery (2009–2014 vs. 2015–2019), and teaching hospital status (yes vs. no).

### 2.4. Statistical Analyses

First, patient and hospital characteristics as well as adverse in-hospital outcomes were tabulated. For continuously coded variables, medians and interquartile ranges (IQR) were recorded. For categorical variables, we recorded frequencies and proportions. Wilcoxon rank sum and Pearson Chi-square tests were used to test for differences between RARP and ORP patients. Second, the estimated annual percentage change (EAPC) for the proportion of the robot-assisted approach within all quadragenarians receiving RP were calculated using least-squares linear regression. Third, multivariable logistic and Poisson regression models predicting adverse in-hospital outcomes were fitted after adjustment for clustering at the hospital level using a generalized estimation equation methodology. Fourth, we performed a sensitivity analysis that relied on propensity score matching (PSM). Specifically, we repeated the comparisons between RARP and ORP outcomes including multivariable analyses after one-to-one PSM between ORP and RARP patients. PSM relied on age at surgery, ethnicity, CCI, PLND, year of surgery, hospital size, and teaching hospital status, according to the nearest neighbor. The objective of PSM was to maximally reduce and ideally eliminate the effects of bias and confounding. The analyses followed the NIS reporting guidelines and counts were censored for samples < 11 [15]. R software was used for statistical computing and graphics (R version 4.3.1, accessed on 16 June 2023, R Foundation for Statistical Computing, Vienna, Austria) [19]. All tests were two-sided with a significance level of *p* < 0.05.

## 3. Results

### 3.1. Overall Characteristics of the Study Population

**Figure 1:**
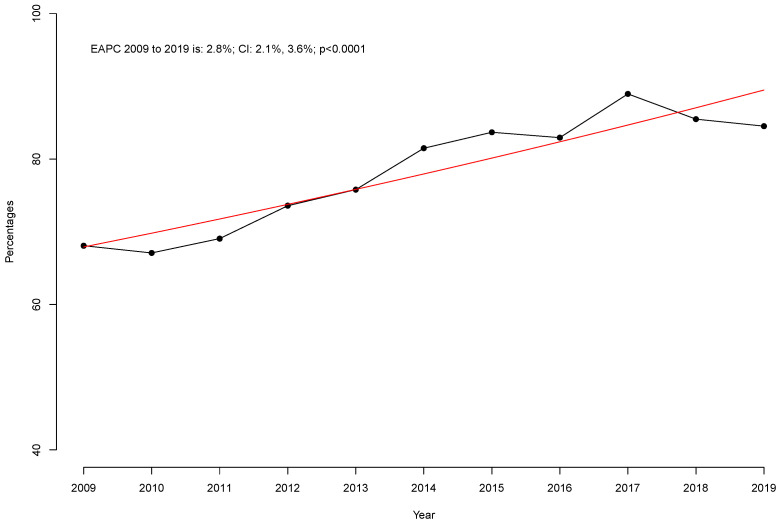
Proportion of robot-assisted approach within all quadragenarians receiving radical prostatectomy for localized prostate cancer over time within the National Inpatient Sample (NIS) from 2009 to 2019. The estimated annual percentage change is visualized as a red line.

Of 133,318 patients undergoing RP for localized PCa between 2009 and 2019 in the NIS, 5426 (4.1%) were quadragenarians. Of these, 4083 (75.2%) and 1343 (24.8%) patients underwent RARP and ORP, respectively (Table 1). From 2009 to 2019, the proportion of RARP use within all quadragenarian RP patients increased from 68.1 to 84.5% (EAPC, +2.8%, *p* < 0.001, Figure 1).

Before PSM, RARP patients less frequently underwent concomitant pelvic lymph node dissection (PLND, 41.2 vs. 44.8%, *p* = 0.02). Moreover, RARP patients were more frequently treated in recent years (2015–2019; 33.0 vs. 17.6%), less frequently in large hospitals (62.8 vs. 68.1%), and more frequently in teaching hospitals (79.4 vs. 67.2%, all *p* < 0.001).

After PSM, of 5426 quadragenarians, 1343 of 1343 (100%) ORP patients and 1343 of 4083 (32.9%) RARP patients remained. After PSM, comparisons addressing patient (age, ethnicity, CCI, PLND, year of surgery) and hospital (large size, teaching status) characteristics resulted in no residual statistically significant differences (Table 1).

### 3.2. Adverse In-Hospital Outcomes Before Propensity Score Matching

Compared to their ORP counterparts, quadragenarian RARP patients exhibited lower rates of adverse in-hospital outcomes in 6 of 16 examined categories (all *p* ≤ 0.02; Table 2). Specifically, RARP was associated with lower rates of overall complications (Δ −5.6%), intraoperative complications (Δ −1.2%), blood transfusions (Δ −5.1%), respiratory complications (Δ −1.2%), genitourinary complications (Δ −0.9%), and LOS > 2 days (Δ −18.1%). Conversely, the median THC was significantly higher after RARP than ORP (Δ USD +8190, *p* < 0.001).

After multivariable adjustment, RARP independently predicted lower rates of adverse in-hospital outcomes in 5 of 16 examined categories (all *p* ≤ 0.01, Table 3). Specifically, RARP use independently predicted lower rates of overall complications (Odds Ratio (OR): 0.54), blood transfusions (OR: 0.21), respiratory complications (OR: 0.43), genitourinary complications (OR: 0.48), and LOS > 2 days (OR: 0.32). Conversely, RARP use independently predicted a 1.20-fold increase in THC (*p* < 0.001).

### 3.3. Adverse In-Hospital Outcomes After Propensity Score Matching

After one-to-one propensity score matching for age, ethnicity, CCI, PLND, year of surgery, hospital size, and teaching hospital status, quadragenarian RARP patients exhibited lower rates of adverse in-hospital outcomes in 5 of 16 examined categories (all *p* ≤ 0.02; Table 2). Specifically, RARP was associated with lower rates of overall complications (Δ −6.4%), blood transfusions (Δ −4.8%), respiratory complications (Δ −1.2%), genitourinary complications (Δ −0.5%), and LOS > 2 days (Δ −17.8%). Conversely, the median THC (Δ USD +6850, *p* < 0.001) was significantly higher after RARP than ORP.

After multivariable adjustment, RARP independently predicted lower rates of adverse in-hospital outcomes in 4 of 16 examined categories (all *p* ≤ 0.02, Table 3). Specifically, RARP use independently predicted lower rates of overall complications (OR: 0.49), blood transfusions (OR: 0.23), respiratory complications (OR: 0.40), and LOS > 2 days (OR: 0.30). Conversely, RARP use independently predicted a 1.18-fold increase in THC (*p* < 0.0001).

## 4. Discussion

We focused on quadragenarian RP patients because this patient group has not been specifically addressed in the existing English language medical literature, but it represents a unique patient population at risk due to several reasons, such as higher baseline urinary and erectile function, longer life expectancy, and different cancer phenotypes. Moreover, patients in their 40s are often at the peak of their careers and may have young families. Therefore, the potential impact of adverse in-hospital outcomes may be particularly devastating for these patients and their families. Additionally, we hypothesized that RARP and ORP exhibit the same adverse in-hospital outcome profile in quadragenarians. The current analyses yielded several noteworthy observations.

First, 5426 of 133,318 RP patients (4.1%) were aged 40–49 years. This cohort of interest, that includes quadragenarian RP patients, is the largest (*n* = 5426) and most contemporary (2009–2019) such cohort ever reported. Only two previous studies specifically focused on complications in quadragenarian RP patients. Both studies included substantially smaller patient populations. Twiss et al. reported on 66 ORP and Labanaris et al. on 68 RARP quadragenarians [5,11]. Both studies were also substantially more historical (pre-dissemination era—Twiss et al., 2000–2003; Labanaris et al., 2006–2010) than the current one (2009–2019). Based on the above observations, the current study represents by far the largest and most contemporary series of young patients who underwent either RARP or ORP.

Second, to the best of our knowledge, the current study provides the largest and only head-to-head comparison between RARP and ORP that exclusively included quadragenarian RP patients. Other investigators have focused on either functional or oncological outcomes of young RP patients. Their results differed remarkable from those of their older counterparts [2,3,4,5,6,8,10,11]. Furthermore, of existing English language literature comparing RARP and ORP, Trinh et al. and Ambrosini et al. also included quadragenarian patients [14,20]. However, no previous study comparing adverse in-hospital outcomes provided age-specific tabulations to determine numbers of quadragenarian RARP vs. ORP patients and their outcomes. In consequence, the current study addresses an existing knowledge gap. This void can only be sufficiently addressed in a contemporary, large-scale population-based study, such as the current one, owing to the relative rarity of quadragenarian RP patients and adverse in-hospital outcomes in these patients.

Third, the proportion of RARP use in quadragenarian RP patients increased from 68.1 in 2009 to 84.5% in 2019 (*p* < 0.001). We are the first to report the increasing importance of RARP for the surgical management of localized PCa in young patients. However, this increase closely parallels the results of studies including all ages without stratification for either young or old RP patients [14,20,21,22,23]. For example, Preisser et al. reported an increased RARP utilization rate from 60.4 in 2009 to 81.5% in 2015 for all men undergoing RP [23].

Fourth, we identified important differences in patient and hospital characteristics that distinguished quadragenarian RARP patients from their ORP counterparts. Specifically, RARP patients exhibited lower rates of pelvic lymph node dissection (41.2 vs. 44.8%). RARP patients were more frequently treated at teaching hospitals (79.5 vs. 67.2%) and less frequently treated at large hospitals (62.8 vs. 68.1%). Finally, RARP surgeries were performed more frequently in recent years, from 2015 to 2019 (33.0 vs. 17.6%, all *p* < 0.001). These patient (PLND) and hospital (size, teaching status, year of surgery) characteristics represent established predictors of more favorable in-hospital outcomes [1,20,24,25,26]. In consequence, detailed multivariable adjustment and ideally additional propensity score matching for these differences are warranted when such population differences distinguish one subgroup from another. These methodologies were universally applied in the current study.

Fifth, we addressed adverse in-hospital outcomes of RARP and ORP in quadragenarians. After detailed multivariable adjustment, RARP was associated with lower rates of overall complications (Δ −5.6%, reduction by −46%), blood transfusions (Δ −5.1%, reduction by −79%), respiratory complications (Δ −1.2%, reduction by −57%), and LOS > 2 days (Δ −18.1%, reduction by −68%). After additional one-to-one propensity score matching between RARP and ORP for patient and hospital characteristics, virtually the same results were recorded, namely, lower rates of overall complications (Δ = −6.4%, reduction by −51%), blood transfusions (Δ −4.8%, reduction by −77%), respiratory complications (Δ = −1.2%, reduction by −60%), and LOS > 2 days (Δ = −17.8%, reduction by −70%). Of note, RARP use was an independent predictor of cardiac complications (increase by 229%), which warrants further investigation and may be explained by some of the RARP requirements, such as steep Trendelenburg positioning or prolonged pneumoperitoneum. However, in general, these findings provide robust proof that RARP offers an adverse in-hospital outcome advantage in quadragenarians. These observations cannot be directly compared with those of existing studies, since no such studies exist. However, they are in agreement with prior studies examining the perioperative morbidity advantages of RARP, which relied on patients across all age categories [14,20,21,22,27,28,29,30].

Sixth, we examined the differences in THC according to RARP and ORP use among quadragenarians. Here, RARP exhibited a 1.20-fold higher THC relative to ORP after multivariable adjustment. Specifically, RARP added on average USD 8190 to THC compared with ORP. Based on the population at hand, 4046 RARP surgeries instead of ORP resulted in a total cost excess of USD 30 million despite longer length of stay and higher overall complications. These observations are in agreement with previous studies examining THC in RARP vs. ORP, which did not focus on quadragenarians and addressed patients of all ages [21,22]. However, RARP use also exhibited a protective effect on complications after the index hospitalization in previous analysis relying on patients of all ages. This resulted in downstream benefits, such as reduced readmission rates and lower post-discharge healthcare use, which ultimately led to indifferent cumulative healthcare costs within one year after surgery, despite higher THC at the initial hospitalization as well [31]. While the current analysis only assessed adverse in-hospital outcomes within the initial hospital stay, a similar observation appears plausible for quadragenarians. In consequence, the initial THC disadvantage of RARP deserves contemplation in light of the multiple, albeit mostly subtle, advantages, when adverse in-hospital outcomes of RARP are considered.

Taken together, the current study provided the largest and most up-to-date cohort of quadragenarian RP patients. RARP use within the population has steadily increased over the study period (from 68.1% in 2009 to 84.5% in 2019). RARP was invariably associated with more favorable in-hospital outcome profile. This advantage predominantly relied on overall complications, blood transfusions, and LOS > 2 days, where the most pronounced absolute and relative differences after multivariable adjustment, with and without additional propensity score matching, were observed. Conversely, these advantages are offset by a small, albeit significant, THC increase by RARP use, which requires concomitant consideration. In consequence, RARP clearly and convincingly distinguished itself from ORP in quadragenarian patients when adverse in-hospital outcomes were examined.

Although a large-scale analysis necessitates the use of retrospective, population-based databases, the present study has inherent limitations that relate to this approach. First, our study shares the limitations of all similar studies that were based on the NIS database and relied on a retrospective data design. Specifically, the NIS relies on administrative data that are subject to coding errors, coding inaccuracies, and missing data. Second, data regarding long-term oncological and functional outcomes as well as general health were not available. Third, information on the pathological stage, PSA, or Gleason sum at surgery was not available, which might have influenced patient selection and complication rates. Fourth, the individual expertise of the surgeons was not available, although adjustments were made for hospital size, hospital case volume, and teaching hospital status. In conclusion, clinical interpretations should be drawn with some caution, because the magnitude of selection bias cannot be objectively quantified due to missing clinical variables.

## 5. Conclusions

Quadragenarians exhibited more favorable adverse in-hospital outcomes after RARP vs. ORP use. These advantages are offset by small, albeit significant, increases in complications and (initial) total hospital charges.

## Figures and Tables

**Table 1:** Descriptive characteristics of quadragenarians receiving radical prostatectomy for localized prostate cancer, stratified according to surgical approach, prior and after propensity score matching (PSM) 1:1 (*n* = 5426).

	Prior PSM			After PSM 1:1		
	ORP (*n* = 1343, 24.8%)	RARP (*n* = 4083, 75.2%)	*p*-Value ^1^	ORP (*n* = 1343, 50.0%)	RARP (*n* = 1343, 50.0%)	*p*-Value ^1^
**Age**, median (IQR)	47 (45, 49)	47 (45, 48)	0.97	47 (45, 49)	47 (45, 49)	0.9
**Ethnicity**, *n* (%)			0.7			0.6
Caucasian	737 (54.9%)	2206 (54.0%)		737 (54.9%)	735 (54.7%)	
Afro-American	304 (22.6%)	974 (23.9%)		304 (22.6%)	287 (21.4%)	
Others	302 (22.5%)	903 (22.1%)		302 (22.5%)	321 (23.9%)	
**CCI**, *n* (%)			0.6			0.3
0	1120 (83.4%)	3380 (82.8%)		1120 (83.4%)	1151 (85.7%)	
1	154 (11.5%)	508 (12.4%)		154 (11.5%)	132 (9.8%)	
≥2	69 (5.1%)	195 (4.8%)		69 (5.1%)	60 (4.5%)	
**PLND**, *n* (%)	601 (44.8%)	1681 (41.2%)	0.02	601 (44.8%)	605 (45.0%)	0.9
**Year of surgery**, *n* (%)			<0.001			0.5
2009–2014	1106 (82.4%)	2735 (67.0%)		1106 (82.4%)	1121 (83.5%)	
2015–2019	237 (17.6%)	1348 (33.0%)		237 (17.6%)	222 (16.5%)	
**Large size hospital**, *n* (%)	915 (68.1%)	2566 (62.8%)	<0.001	915 (68.1%)	936 (69.7%)	0.4
**Teaching hospital**, *n* (%)	903 (67.2%)	3247 (79.5%)	<0.001	903 (67.2%)	931 (69.3%)	0.3

^1^ Wilcoxon rank sum test, Pearson’s chi-squared test. Abbreviations: ORP = open radical prostatectomy, RARP = robot-assisted radical proctectomy, IQR = interquartile range, CCI = Charlson Comorbidity Index, PLND = pelvic lymph node dissection.

**Table 2:** Adverse in-hospital outcomes after radical prostatectomy for localized prostate cancer in quadragenarians, stratified according to surgical approach, prior and after propensity score matching (PSM) 1:1. (*n* = 5426).

	Prior PSM				After PSM 1:1			
	ORP (*n* = 1343, 24.8%)	RARP (*n* = 4083, 75.2%)	Absolute Difference RARP vs. ORP (Δ)	*p*-Value ^1^	ORP (*n* = 1343, 50.0%)	RARP (*n* = 1343, 50.0%)	Absolute Difference RARP vs. ORP (Δ)	*p*-Value ^1^
**Overall complications**, *n* (%)	180 (13.4%)	318 (7.8%)	−5.6%	<0.001	180 (13.4%)	94 (7.0%)	−6.4%	<0.001
**Intraoperative complications**, *n* (%)	49 (3.6%)	98 (2.4%)	−1.2%	0.02	49 (3.6%)	37 (2.8%)	−0.8%	0.2
**Critical care therapy**, *n* (%)	<11	15 (0.4%)	−0.1%	0.9	<11	<11	0.0%	0.99
**Bleeding complications**, *n* (%)	16 (1.2%)	33 (0.8%)	−0.4%	0.3	16 (1.2%)	13 (1.0%)	−0.2%	0.7
**Blood transfusions**, *n* (%)	85 (6.3%)	51 (1.2%)	−5.1%	<0.001	85 (6.3%)	20 (1.5%)	−4.8%	<0.001
**Cardiac complications**, *n* (%)	<11	30 (0.7%)	+0.4%	0.1	<11	12 (0.9%)	+0.6%	0.08
**Respiratory complications**, *n* (%)	25 (1.9%)	27 (0.7%)	−1.2%	<0.001	25 (1.9%)	<11	−1.2%	0.02
**Genitourinary complications**, *n* (%)	22 (1.6%)	30 (0.7%)	−0.9%	0.005	22 (1.6%)	15 (1.1%)	−0.5%	0.3
**Wound complications**, *n* (%)	<11	<11	−0.1%	0.3	<11	<11	−0.1%	0.99
**Infectious complications**, *n* (%)	<11	<11	+0.1%	0.5	<11	<11	0.1%	0.99
**Vascular complications**, *n* (%)	<11	<11	−0.1%	0.3	<11	<11	−0.1%	0.6
**Misc. surgical complications**, *n* (%)	<11	19 (0.5%)	−0.1%	0.7	<11	<11	−0.4%	0.2
**Misc. medical complications**, *n* (%)	28 (2.1%)	70 (1.7%)	−0.4%	0.4	28 (2.1%)	15 (1.1%)	−1.0%	0.07
**In-hospital mortality**, *n* (%)	0	0	0		0	0	0	
**Prolonged LOS** (>2 days) †, *n* (%)	385 (28.7%)	432 (10.6%)	−18.1%	<0.001	385 (28.7%)	146 (10.9%)	−17.8%	<0.001
**THC**, USD, median (IQR)	36,840 (24,100, 53,280)	45,030 (32,850, 63,830)	+8190	<0.001	36,840 (24,100, 53,280)	43,690 (31,200, 61,940)	+6850	<0.001

^1^ Wilcoxon rank sum test, Pearson’s Chi-square test. Abbreviations: ORP = open radical prostatectomy, RARP = robot-assisted radical proctectomy. IQR = interquartile range, LOS = length of stay, THC = total hospital charges. † Exceeding the third quartile for the cohort.

**Table 3:** Multivariable regression models predicting adverse in-hospital outcomes according to the surgical approach in quadragenarians undergoing radical prostatectomy for localized prostate cancer, after adjustment for clustering at the hospital level using generalized estimation equation (GEE) methodology, prior to and after propensity score matching (PSM) 1:1 (*n* = 5426).

	Prior PSM		After PSM 1:1	
	Multivariable OR/IRR (95% CI) *	*p*-Value	Multivariable OR/IRR (95% CI) *	*p*-Value
Overall complications	0.54 (0.44–0.67)	<0.001	0.49 (0.38–0.64)	<0.001
Intraoperative complications	0.77 (0.53–1.11)	0.2	0.77 (0.49–1.20)	0.2
Critical care therapy	0.91 (0.32–2.59)	0.9	0.99 (0.32–3.02)	0.99
Bleeding complications	0.76 (0.41–1.40)	0.4	0.83 (0.40–1.74)	0.6
Blood transfusions	0.21 (0.15–0.31)	<0.001	0.23 (0.14–0.37)	<0.001
Cardiac complications	2.02 (0.68–6.03)	0.2	3.29 (1.02–10.63)	0.047
Respiratory complications	0.43 (0.24–0.77)	0.005	0.40 (0.19–0.84)	0.02
Genitourinary complications	0.48 (0.27–0.85)	0.01	0.69 (0.37–1.28)	0.2
Wound complications	0.30 (0.06–1.49)	0.1	0.68 (0.11–4.39)	0.7
Infectious complications	1.98 (0.29–13.32)	0.5	2.34 (0.34–16.00)	0.4
Vascular complications	0.29 (0.07–1.22)	0.09	0.36 (0.04–3.29)	0.4
Misc. surgical complications	0.94 (0.42–2.10)	0.9	0.38 (0.10–1.43)	0.2
Misc. medical complications	0.68 (0.43–1.08)	0.1	0.58 (0.31–1.08)	0.09
Prolonged LOS (>2 days) †	0.32 (0.27–0.38)	<0.001	0.30 (0.24–0.37)	<0.001
Total hospital charges	1.20 (1.15–1.25)	<0.001	1.18 (1.13–1.24)	<0.001

Reference: open radical prostatectomy. * adjusted for patient age at surgery, Charlson Comorbidity Index, year of surgery, PLND, hospital size, and teaching hospital status. † Exceeding the third quartile for the cohort. Abbreviations: OR = odds ratio, IRR = incidence rate ratio, CI = confidence interval, LOS = length of stay.

## Data Availability

These data were derived from the National Inpatient Sample (NIS) database in the public domain: https://hcup-us.ahrq.gov/databases.jsp, accessed on 31 May 2024.
